# Structural Characterisation of Deposit Layer during Milk Protein Microfiltration by Means of *In-Situ* MRI and Compositional Analysis

**DOI:** 10.3390/membranes10040059

**Published:** 2020-03-31

**Authors:** Roland Schopf, Nicolas Schork, Estelle Amling, Hermann Nirschl, Gisela Guthausen, Ulrich Kulozik

**Affiliations:** 1 Chair of Food and Bioprocess Engineering, Technical University of Munich, Weihenstephaner Berg 1, 85354 Freising, Germany; ulrich.kulozik@tum.de; 2 Institute of Mechanical Process Engineering and Mechanics, Karlsruhe Institute of Technology (KIT), 76131 Karlsruhe, Germany; estelle.amling@gmail.com (E.A.); hermann.nirschl@kit.edu (H.N.); gisela.guthausen@kit.edu (G.G.); 3 Chair of Water Chemistry and Water Technology, Karlsruhe Institute of Technology (KIT), 76131 Karlsruhe, Germany

**Keywords:** fouling, fractionation, casein, whey protein, filtration resistance

## Abstract

Milk protein fractionation by microfiltration membranes is an established but still growing field in dairy technology. Even under cross-flow conditions, this filtration process is impaired by the formation of a deposit by the retained protein fraction, mainly casein micelles. Due to deposition formation and consequently increased overall filtration resistance, the mass flow of the smaller whey protein fraction declines within the first few minutes of filtration. Currently, there are only a handful of analytical techniques available for the direct observation of deposit formation with opaque feed media and membranes. Here, we report on the ongoing development of a non-invasive and non-destructive method based on magnetic resonance imaging (MRI), and its application to characterise deposit layer formation during milk protein fractionation in ceramic hollow fibre membranes as a function of filtration pressure and temperature, temporally and spatially resolved. In addition, the chemical composition of the deposit was analysed by reversed phase high pressure liquid chromatography (RP-HPLC). We correlate the structural information gained by *in-situ* MRI with the protein amount and composition of the deposit layer obtained by RP-HPLC. We show that the combination of in-situ MRI and chemical analysis by RP-HPLC has the potential to allow for a better scientific understanding of the pressure and temperature dependence of deposit layer formation.

## 1. Introduction

Milk protein fractionation by microfiltration (MF) membranes is a still growing field in dairy technology and a lead technology for the valorisation of complex food materials such as milk by making single fractions available with their unique individual functional properties. It is generally known that, in membrane filtration, a considerable accumulation of retained material on the membrane surface occurs, in particular during milk protein fractionation by MF.

During MF of food systems, material accumulations at the membrane surface mainly consist of biopolymers such as proteins or polysaccharides [1,2,3,4]. This applies even under crossflow conditions, where the wall shear stress only reduces the amount of deposited material, but a complete prevention of material accumulation cannot be achieved [5,6,7]. This is referred to deposit formation [8]. In applications in dairy technology, the deposit primarily consists of casein micelles, the main protein component in milk [9,10,11,12]. Previous studies showed that the total filtration resistance considerably increases as a function of time and processing conditions owing to the deposit layer formation and cross-linking of proteins in the deposit, which leads to a steep decline in flux with filtration time in the very first minutes after filtration started [13,14,15,16,17]. In protein fractionation by MF, deposit formation by the fraction of the larger proteins (casein micelles) has a decisive negative effect since it hinders the desired permeation of the smaller whey proteins [18]. Therefore, the deposit layer formation correlates with a loss in separation efficiency and throughput [4]. This is not only of relevance for the overall fractionation result of the whole module. Since in cross-flow membrane filtration the transmembrane pressure decreases along the flow path from module inlet to outlet, the extent of deposit formation varies accordingly, while the shear stress almost stays the same as at the inlet. As found by Hartinger et al. [5], Piry et al. [19], and Kulozik & Kersten [4] using custom-made module systems able to measure flux and permeate composition in segregated modules separately, the targeted maximum of whey protein permeation is achieved only at or towards the end of the module. At this point, the ratio of transmembrane pressure and shear stress shifts to the benefit of shear forces imposed on already deposited or depositing material. Deposit formation is thus reduced where the transmembrane pressure is gradually reaching lower levels, while shear forces transporting deposited material away from the membrane surface stay the same (if one neglects the minimal amount of permeate reducing the volume flow from module inlet to outlet). Kühnl et al. [16] and Steinhauer et al. [20] have further extended these insights by indirectly studying the effect of colloidal interactions between particles in the deposited layer at the membrane. There is room for optimizing the efficiency of, e.g., milk protein fractionation by creating more or less open porous deposits as a result of varying attractive or repulsive forces between particles as a function of pH and ionic strength. It would be of interest to be able to assess these structural details *in-situ*, but a capable method does not yet exist.

To investigate the accumulated material with regard to their chemical or structural properties, indirect or destructive techniques are widely used. Because the deposit layer changes its appearance, composition and density upon pressure release, the deposit layer should ideally be investigated *in-situ* and non-destructively. Furthermore, changes in height and inner structure are expected because the deposited layer expands due to relaxation under ambient pressure conditions. In addition, ex-situ measurements require the removal and destruction of the membrane in order to get access to the deposited material.

In comparison to previously investigated clear model fluids multiple complications arise: (1) milk is an in-transparent fluid and (2) membrane systems, such as tubular ceramic or polymeric hollow fibres that are operated in inside-out filtration mode, are difficult to be analysed by direct observation methods, e.g., by optical means.

In previous studies [21,22,23,24,25,26], nuclear magnetic resonance imaging (MRI) was used as a non-invasive and non-destructive technique to assess the growth of a deposit layer during filtration of diverse materials as a function of filtration time. These works mostly studied model systems consisting of inorganic particles or defined hydrocolloids such as alginate in aqueous solution. Skim milk as an in-transparent feed medium and especially the deposit layer formation in ceramic hollow fibre membranes was also studied [27,28] to prepare the methodology of the MRI measurements. MRI was applied on skim milk as a complex medium and to establish the required sample preparation to achieve the necessary contrast difference for MRI analysis and to develop the mathematical data processing protocol. When using MRI, spatially and time-resolved information about density and height of the deposit layer can therefore be obtained for skim milk filtration in inaccessible, optically in-transparent module housings to directly and *in-situ* assess deposit formation.

Beyond this, membrane filtration performance depends on the processing conditions such as pressure, temperature and filtration time. However, it is unclear whether the time-dependent filtration performance depends on the effect of the change in deposit height because of protein accumulation or change in porosity due to deposit compaction. When trying to better understand these effects and changes of the deposit layer it is obvious that this gap in knowledge needs to be filled to explain the impact of deposit layer formation on flux reduction. Furthermore, an answer to questions regarding the impairment of permeation of solutes through the combined layers of membrane and deposit material is of interest because the deposit layer acts as an additional filtration resistance in the form of a secondary membrane.

Regarding processing conditions it is known that a loss in static pressure not only occurs along the flow path (Δp_L_), i.e., along a membrane, but also inside the deposited material when the permeate flows towards the membrane surface because of frictional pressure losses, thus reducing the transmembrane pressure (Δp_TM_) [29]. For a layer of compressible and deformable casein micelles, the pressure loss results in an increase in compaction and, thus, in higher deposits´ densities or reduced porosities [20]. A reduction of flux with filtration time during MF of whey proteins has been attributed to an increase of the deposit layer height alone [30]. Apart from that, some studies were performed to qualitatively and quantitatively elucidate the structure of the deposit [31,32,33,34,35,36]. However, a correlation between the chemical analysis of the deposit layer and *in-situ* measured layer has not been shown so far. The methods developed in [26,27,28] are applied to get more insight into the details of the deposit´s composition regarding the ratio of casein and whey protein. Additionally, there is still a lack of understanding of how pressure affects the behaviour of the deposit’s chemical composition and height.

Apart from Δp_L_ and Δp_TM_, temperature *ϑ* is a variable in membrane filtration that results in increasing flux and, therefore, a higher convective transport of protein towards the membrane surface at higher temperatures. Its influence on deposit formation and filtration efficiency is still the subject of current research [37]. In industrial applications, the MF process of skim milk is operated either at rather low *ϑ* (10–15 °C) [38] or rather high temperatures of about 45–55 °C [39]. Higher temperatures yield higher fluxes mainly due to the lower product viscosity, but may promote denaturation and also microbial growth to some extent, especially in long-term operations between cleaning cycles [1]. Meanwhile, lower temperatures keep microbial growth better under control, but yield low fluxes and induce a migration of β-casein into the permeate, thus reducing the purity of the whey protein fraction [40,41]. Despite the fact that cold filtration is by now more frequently applied in industrial application, little is known about the effect of low temperature in milk MF on the deposition of casein micelle layers.

Many models exist that try to determine the filtration phenomena by evaluating the macroscopic data such as the permeate flux or pressure drop [42]. The deposit formation is affected by more than only one of these effects: pore blocking, pore constriction, cake formation, solute adsorption and concentration polarization [43]. In case of the *in-situ* measurement by MRI the deposit formation during skim milk filtration is observed on a microscale where it was already shown that parts of the deposit layer behave differently as the pressure is released as a part of it diffuses back into the membrane lumen [25].

In summary, the purpose of this study is to apply MRI *in-situ* to assess milk protein deposits on MF membranes applied for milk protein fractionation under processing conditions relevant for practical situations. MRI is currently in ongoing development to be applied in various filtration processes. MF was at first operated in dead-end mode to measure the impact of Δp_TM_, filtration time, and filtration temperature. To correlate MRI results with chemical composition and the permeation of proteins, reversed-phase high-performance liquid chromatography (RP-HPLC) analysis of the deposit layer was performed. These data allow a compositional characterisation of the deposit layer and could thus lead to a deeper understanding of mechanisms responsible for the reduction of flux and protein permeation caused by deposited materials on membrane surfaces.

## 2. Materials and Methods

### 2.1. Reconstituted Skim Milk Solution

The skim milk solution was produced by redispersion of ‘low heat skim milk powder’ from ‘milk and whey ingredients’, Sachsenmilch Leppersdorf GmbH, Germany. The composition of the reconstituted skim milk is listed in Table 1. 103.2 g of the skim milk powder was dissolved in *V*_water_ = 1 L of demineralised water, which resulted in 2.8 %w/w of casein, a total protein content of 3.5 %w/w and pH 6.6, as is typical for bovine milk [44,45]. All experiments were performed at room temperature (*ϑ* = 22 °C). Viscosities of water and permeate were measured with an Anton Paar MCR 302 rheometer (Anton Paar, Graz, Austria) using the double-gap geometry. The rehydrated skim milk powder is referred to as skim milk hereafter [23].

### 2.2. Ceramic Hollow Fibre Membranes

The ceramic hollow fibre membranes, provided by MANN + HUMMEL GmbH, Ludwigsburg, Germany, were operated in inside-out filtration mode. The selective layer of α-Al_2_O_3_ was on the inner surface of the channel. The hollow fibre has an inner diameter of *d*_i_ = 1.9 mm and an outer diameter of *d*_o_ = 3.2 mm. The nominal average pore diameter, as stated by the manufacturer, is approximately 100 nm. The hollow fibre membranes have an average pure water permeability of *J*_0_ = 438.5 L m^−2^ h^−1^ bar^−1^ and a membrane resistance *R*_M_ of 8.28·10^11^ m^−11^. One single hollow fibre membrane was used in the MRI measurements for each filtration pressure and temperature.

### 2.3. MRI Contrast Agent

**Figure 1:**
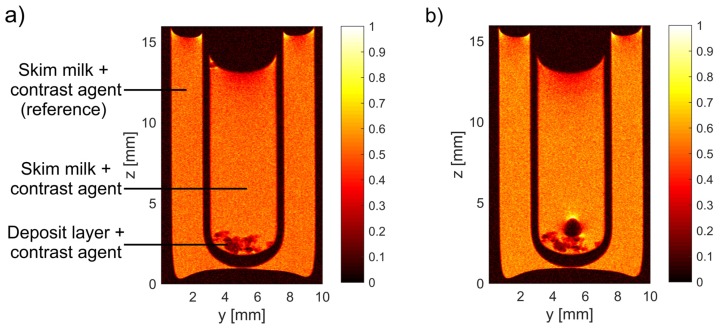
MIONs are embedded into the deposit layer during filtration. (**a**) After filtration, the deposit was taken out of the hollow fibre and placed into a double glass tube filled with feed. (**b**) The nanoparticles are still embedded in the deposit after *t* = 53 h and do not diffuse back into the surrounding feed. Only a gas bubble formed on the deposit layer. If the contrast agent had diffused back into the souring skim milk, a difference in the signal intensity to the reference solution would have been measured.

A contrast agent in the form of magnetic iron oxide nanoparticles (MIONs) with a concentration of *c*_Fe_ = 4.26·10^−3^ mM_Fe_ (corresponding to a concentration of 0.024 %w/w) was added to the feed solution before filtration. The protein deposit on the membrane can also be measured exploring the native MRI contrast, i.e., without contrasting agent. However, a better contrast between feed and deposit is achieved because of the high transverse relativity of MIONs [25]. Lower signal intensities are induced because of the dominantly transverse paramagnetic relaxation enhancement of the nanoparticles. The contrast agent used in the experiments, commercially available as ‘nanomag-D-spio’, was purchased from micromod Partikeltechnologie GmbH, Germany. The MIONs with a mean diameter of 100 nm are composed of iron oxide clusters embedded in a matrix of dextran and are coated with casein proteins to be chemically compatible with the feed. In previous studies [21,22,23,24,25,27,28], it was shown that the contrast agent is embedded in the deposit layer and does not diffuse back into the feed solution separately from the deposit nor significantly permeates the membrane. To confirm and investigate the behaviour of these MIONs, the deposit layer was taken out of the hollow fibre membrane after filtration with MIONs and put into a glass tube filled with skim milk, also including the contrast agent. For direct comparison, a second glass tube with a reference skim milk was placed around the inner tube (Figure 1).

### 2.4. In-situ MRI

**Figure 2:**
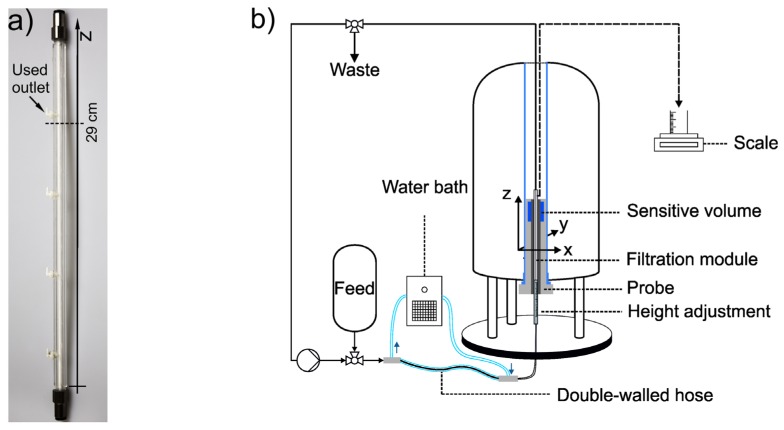
(**a**) The ceramic hollow fibre membrane was placed into an MRI-compatible filtration module for *in-situ* MRI. The permeate outlet at *z* = 32 cm was used and an axial slice at *z* = 29 cm was measured directly underneath the permeate outlet. (**b**) Scheme of the setup for the *in-situ* filtration. The hollow fibre module is connected to a filtration rig. A double-walled hose with a counter-current flow system is integrated before the membrane module to enable measurements at different skim milk temperatures. Additionally, the temperature of the gradient system inside the magnet bore, directly around the MRI probe is adjusted accordingly.

The *in-situ* MRI measurements were performed by implementing the filtration module containing the membrane in a Bruker Avance HD III SWB 200 MHz spectrometer (Bruker Biospin GmbH Rheinstetten, Germany) [22,24,28] (Figure 2).

The filtration module was attached to the adjustment rod with an inscribed length scale that can be carefully set to the measurement point along the *z*-axis of the hollow fibre, in this case at *z* = 29 cm, just below the permeate outlet [24]. The filtration module was connected to a filtration rig and placed into a 20 mm birdcage of the MICWB40 series.

Skim milk filtrations monitored by MRI were performed in dead-end filtration mode at three different feed pressures *p* = 0.25, 0.75, and 1.25 bar. Because no permeate back pressure was applied to the filtration system the transmembrane pressure is equivalent to the feed pressure in the dead-end filtration mode. To investigate the temperature-dependent deposit formation, the filtration was also performed at three different temperatures (*ϑ* = 15, 22, and 45 °C). The highest temperature of 45 °C was chosen with a small safety window because the gradient cooling system has an overheating protection at 50 °C. To ensure a fully tempered skim milk, the feed solution was thermostated while stirred either with a heating plate or precooled in a refrigerator. To prevent a heat loss during filtration, the skim milk was kept at the desired temperature by a double-walled hose system. The skim milk was fed into the inner hose (2.5 mm diameter) while distilled water streams in counter-current flow through the outer hose (10 mm diameter) that was tempered to the desired temperature by a Thermo Scientific DC30 thermostat. During filtration, the temperature of the magnetic field gradient system, in which the imaging birdcage is installed, was additionally set to the desired temperature to ensure a minimal temperature gradient. Additionally, all inlet tubes were thermally insulated to keep the temperature loss of the milk as low as possible. After the experiments, the temperature of the milk in the module was measured with a PT100 thermometer. A deviation of about 2 °C from the set temperature was measured. It should be noted that it is only possible to measure the milk temperature after the module has been removed from the *in-situ* MRI setup for which the pressure needs to be slowly released and the imaging probe containing the filtration module carefully removed from the magnet system. Afterwards, the insulation needs to be taken off before a temperature sensor can be inserted into the module, which takes a few minutes leading to an increase of the temperature deviation.

During the first 20 to 30 min of the dead-end filtration, the measured flux rapidly declined until nearly a steady-state was reached. A “Rapid Acquisition with Relaxation Enhancement” (RARE) MRI pulse sequence was chosen. The measurements were acquired with a time resolution of approximately 5 min. MR measurements are known to be susceptible to flow. The repetition time *T*_R_ needs to be set sufficiently long and the RARE factor (RF) needs to be low to prevent inacceptable large flow artefacts (Table 2). The measurement time of 5 min 8 s for MRI data is set sufficiently short to measure the deposit formation during filtration while still allowing an almost artefact-free quantification of the acquired data.

### 2.5. MR Image Data Processing

The deposit layer is radially uniformly distributed on the membranes’ inner surface, which is evidenced by the MR images. However, the ceramic hollow fibres are not always perfectly round owing to the manufacturing process. Therefore, before filtration, a reference MR image was acquired in which the membrane was filled with water. This allows an accurate definition of the geometry for the segmentation of the membrane lumen from the hollow fibre membrane via an intensity threshold. An equivalent channel diameter (Equation (1)) was defined by the segmented lumen area *A*_Feed_:

(1) $$d_{\text{equiv.}}={\left(4\cdot \frac{A_{\mathrm{Feed}}}{\pi }\right)}^{\frac{1}{2}}.$$

To process the MR images quantitatively, a self-written MATLAB script was used to determine the intensity in the lumen and determine the mean height of the deposit layer [24,25]. A mask is defined with the exact shape of the membrane lumen, which enables the processing of not perfectly round or even arbitrarily shaped lumen geometries.

In the MR images acquired during filtration, the deposit can be distinguished from the feed area via a user-defined intensity threshold. The equivalent diameter of the segmented inner lumen mask, which represents the feed area, is calculated according to Equation (1). The determined reference and deposit equivalent diameters are used to finally calculate the average deposit thickness *h*_d_ (Equation (2)):

(2) $$h_{d}=\frac{\left(d_{\text{equiv.}}\left(\mathrm{reference}\right)-d_{\text{equiv.}}\left(\mathrm{deposit}\right)\right)}{2}.$$

The quantification of *h*_d_ is similar to the method used in [22]. A second intensity threshold is applied instead of a second discrete ellipse. Instead of the ellipse diameters, the equivalent diameters [Equation (1) and Equation (2)] are utilized to quantify the deposit height.

### 2.6. Analysis of the Protein Composition of the Deposit Layer

To correlate MRI and RP-HPLC results, the same pieces of the deposit layer in the hollow fibre membrane measured by MRI (deposit height) were analysed by RP-HPLC (protein composition and amount). To assess the protein composition, the hollow fibre was cut into accurately defined pieces with a length of Δ*z* = 1 cm at each MRI *z* measurement position. Then, the cut hollow fibre was placed in 2 mL of a guanidine buffer based on Bonizzi [46] (Tris(hydroxymethyl)aminomethane (TRIS, 0.165 mol L^−1^), urea (8.000 mol L^−1^), sodium-citrate (0.044 mol L^−1^), and β-mercaptoethanol (0.3% *v/w*)). The buffer removed and dissolved the deposited proteins from the hollow fibre membrane. Next, the samples with the dissolved deposit layer were filtrated (0.45 µm) into vials. Casein fractions of α_S1_, α_S2_, β, and κ-casein as well as the whey protein fractions of α-lactalbumin and β-lactoglobulin A and B were quantitatively analysed by RP-HPLC using the method described by Dumpler et al. in [47]. In brief, the method works as follows: the separation of the milk proteins was carried out with an Agilent Zorbax 300SB-C18 150 × 4.6 mm resin with a pre-column (Agilent Technologies, Böblingen, Germany) and the gradient elution (Eluent A, 10% acetonitrile (1 mL L^−1^ trifluoroacetic acid)). Eluent B, 90% acetonitrile (0.7 mL L^−1^ trifluoroacetic) was carried out at *ϑ* = 40 °C and *v* = 1.2 mL min^−1^. The injection volume of the samples with the dissolved deposit layer was 100 µL. UV–VIS absorption was measured at a wavelength of 226 nm.

## 3. Results and Discussion

### 3.1. MRI Measurements of Deposit Layer

**Figure 3:**
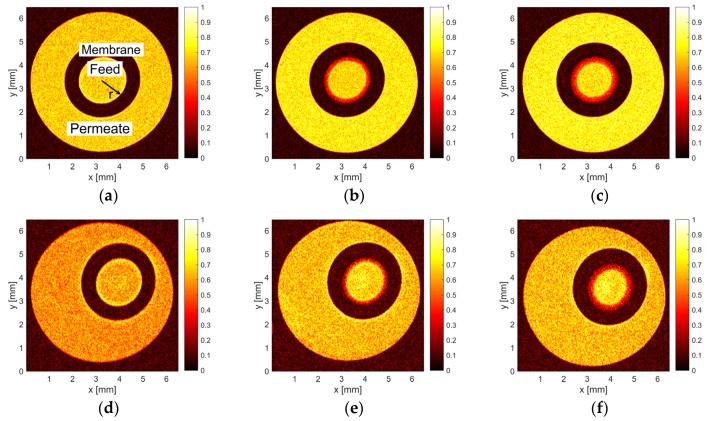
Axial MR images were measured every 5 min at *z* = 29 cm during dead-end filtration at *p* = 1.25 bar and different temperatures *ϑ* = 15 °C (**a**–**c**) and *ϑ* = 45 °C (**d**–**f**). The normalised MR intensity *I*/*I*_max_ is indicated in the false-colour bar. (**a**,**d**) A reference image of the unused hollow fibre was acquired before each filtration. (**b**) Build-up of the protein deposit at *t* = 79 min of filtration (red ring at the inner surface of the membranes lumen) and *t* = 151 min (**c**) for the lowest temperature *ϑ* = 15 °C. Progressing accumulation of the deposit was observed on the selective layer of the hollow fibre membrane at *t* = 67 min (**e**) and 168 min (**f**) during the dead-end filtration at *ϑ* = 45 °C.

MR images of the retained skim milk components were measured during dead-end filtration. Filtrations with three different feed pressures and at three different temperatures were carried out while multiple MR images were acquired as a function of filtration time. During all experiments, the deposit on the inner surface of the hollow fibre increased as the filtration time progressed (Figure 3). The short time required for the measurement of an MR image (Table 2) allows an adequate time resolution to observe the deposit formation. A progressing accumulation leads to an increase in filtration resistance, which causes a rapid decline in the permeate flux, i.e., water and whey protein permeation.

At higher temperatures, the NMR signal-to-noise ratio is lower due to the Curie law (compare Figure 3a–c and Figure 3d–f) [48]. Despite of the lower signal-to-noise ratio, a quantification of the intensities was still possible. A lower normalised intensity *I*/*I*_max_ at the inner surface of the membranes (Figure 3) indicates a higher concentration of retained MIONs and proteins because both, the proteins and contrast agent induce a lower MR signal intensity. Therefore, normalised signal intensities *I*/*I*_max_ decrease as a function of radius *r* at a given filtration time, which indicates the deposit formation when measured as a function of filtration time. While increasing pressure, the steep decrease in *I*/*I*_max_ appears at smaller radii at a given filtration time, i.e., the deposit height is slightly larger.

### 3.2. Compositional Analysis of the Deposit Layer

**Figure 4:**
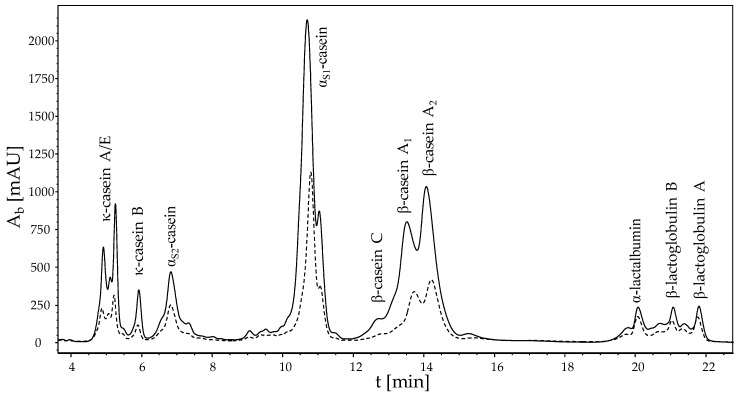
RP-HPLC chromatogram of the deposit (solid line) and the retentate skim milk (dashed line) of caseins and whey proteins in a guanidine buffer at *z* = 29 cm after approximately 3 h of dead-end filtration at *p* = 1.25 bar and *ϑ* = 22 °C. An enrichment of the different caseins in the filtration deposit is evident.

To analyse the protein composition of the deposit layer after filtration, the hollow fibre was cut into a small membrane segment with a length of Δ*z* = 1 cm at the *z* measurement position of the MRI. Figure 4 shows an RP-HPLC chromatogram of the dissolved deposit and the retentate skim milk of caseins and whey proteins at *z* = 29 cm.

All genetic variants of casein and individual whey proteins were detected and analysed. The chromatogram shows that primarily casein deposited on the membrane surface and formed the deposit layer. As expected, the milk proteins are highly concentrated on the surface. By calculating the amount of proteins deposited on the membrane surface, we identified *c_protein_* = 120.1 g m^−2^ of casein and *c_protein_* = 11.0 g m^−2^ of whey proteins on the hollow fibre membrane at the end of the dead-end filtration (≈3 h). In other words, there is about 11 times more casein than whey protein on the surface. This can be attributed to the fact that the diameter of the caseins (50–400 nm) is larger than the pore size of the membrane with 0.1 µm and are therefore preferentially retained. Although whey proteins are significantly smaller than caseins with a mean diameter of 8 nm, they do not permeate the membrane to 100%. They are also concentrated in the deposit. This can be explained by the additional retention effect of the deposited caseins. This retention effect has also an impact on the filtration performance.

### 3.3. Influence of Pressure on Flux

**Figure 5:**
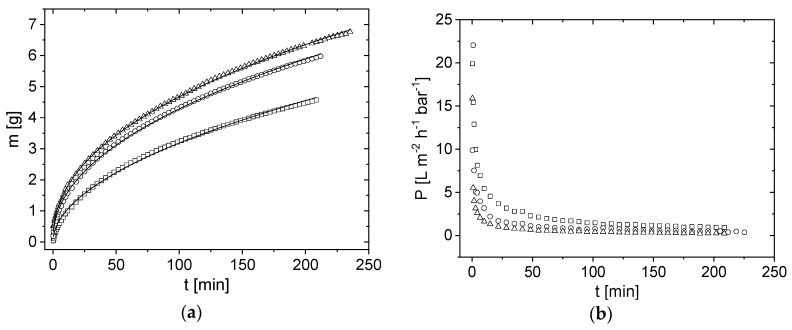
(**a**) Permeate mass *m* as a function of filtration time *t* for the three different feed pressures *p* = 0.25 bar (squares), *p* = 0.75 bar (circles) and *p* = 1.25 bar (triangles) at *ϑ* = 22 °C. Each membrane has a slightly different pure water permeability, which is insignificant on the present scale and therefore neglected here. The integrally measured permeate mass *m*(*t*) was described empirically by *m*(*t*) ∝ *t^b^*^,integral^ (lines) [25]. (**b**) The integral permeability *P* = *J*/*p* depends on the filtration pressure.

**Figure 6:**
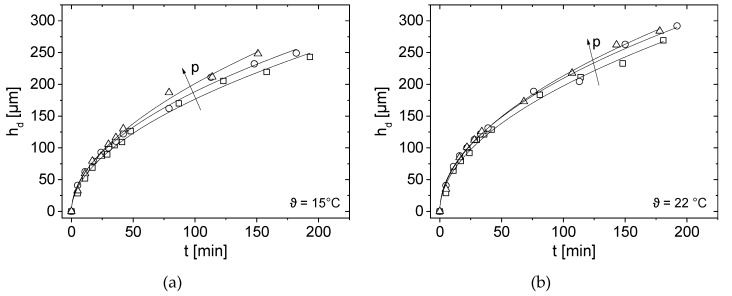

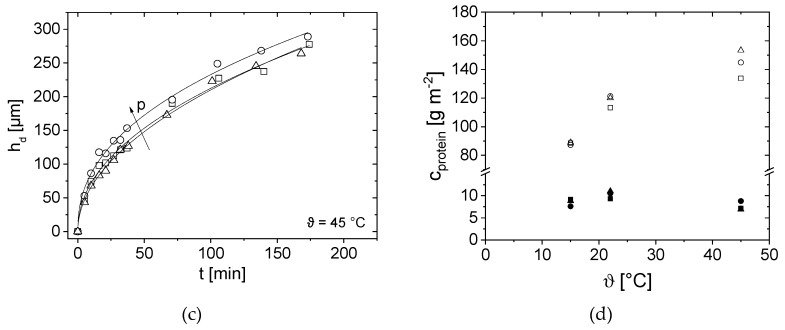
Deposit height *h*_d_ at *z* = 29 cm as a function of filtration time *t* for (**a**) *ϑ* = 15 °C (**b**) *ϑ* = 22 °C and (**c**) *ϑ* = 45 °C. A small difference in deposit height was observed especially at large filtration times for *p* = 0.25 bar (squares), *p* = 0.75 bar (circles) and *p* = 1.25 bar (triangles). The deposit height was empirically modelled by *h*_d_(*t*) ∝ *t^b^*^,local^ (lines). (**d**) Protein concentration *c_protein_* on the membrane surface as a function of filtration temperature *ϑ* for *p* = 0.25 bar (squares), *p* = 0.75 bar (circles) and *p* = 1.25 bar (triangles). Open symbols represent caseins and closed whey proteins.

**Figure 7:**
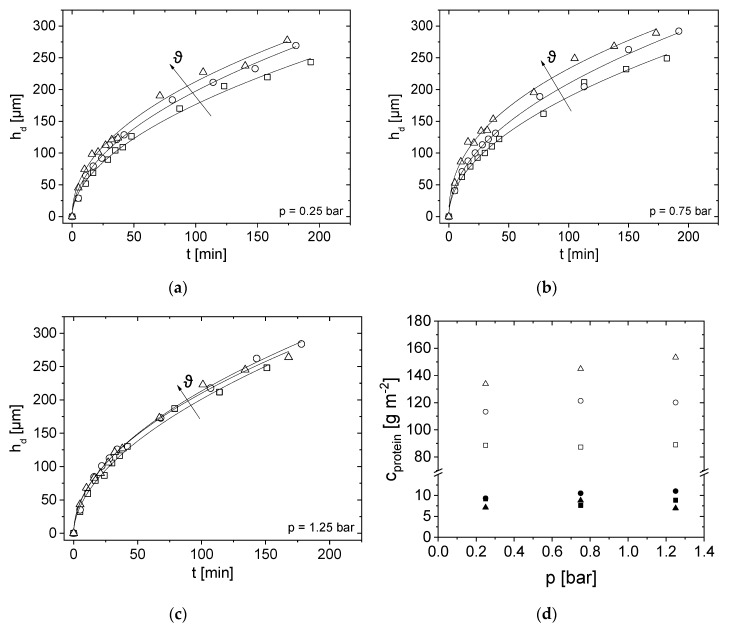
*h*_d_ at *z* = 29 cm as a function of filtration time *t* at (**a**) *p* = 0.25 bar (**b**) *p* = 0.75 bar and (**c**) *p* = 1.25 bar. Temperature was set to *ϑ* = 15 °C (squares), *ϑ* = 22 °C (circles) and *ϑ* = 45 °C (triangles). (**d**) Protein concentration *c_protein_* for caseins and whey proteins on membrane surface as a function of the pressure *p* for the three temperatures for *p* = 0.25 bar (squares), *p* = 0.75 bar (circles) and *p* = 1.25 bar (triangles). Open symbols are casein and closed are whey proteins.

In dead-end filtration mode, the integral quantities mass and permeability *P* (Figure 5) as well as the deposit height *h*_d_ on the membrane surface (Figure 6 and Figure 7) were measured. *h*_d_ increased as a function of time at all measured pressures and temperatures (*p*,*T*). The deposit height increased rapidly at the start of filtration and flattened slightly over time. As the deposit layer grew, the filtration resistance increased with filtration time, resulting in a lower permeate flux. The convective flow is more hindered and the deposit layer therefore increased progressively slower. Specifically, the change in *h*_d_ was at its maximum at the beginning of the filtration because the highest amount of the deposit was transported towards the membrane surface. In the first few minutes of filtration, the integrally measured flux rapidly declined from the pure water flux *J*_0_ to a quasi-stationary flux, which was significantly reduced compared to the initial flux.

The integral permeability *P* of the module was calculated as *P* = *J*/*p*, where *J* is the flux and *p* is the applied pressure. As expected (Figure 5b), *P* decreases with filtration time, starting with the pure water permeability at *t* = 0 min. In addition, the steepness of *P(t)* depends on the filtration pressure. The permeability of the filtration with the highest pressure at *p* = 1.25 bar decreases faster than that at a lower pressure (Figure 5b).

### 3.4. Effect of Pressure and Temperature on Deposit Layer Height

Experimental results are compared at the three different pressures for a given temperature (Figure 6) and then vice versa (Figure 7) to allow an easier interpretation of the influence of *p* and *ϑ*. The deposit thickness *h*_d_ according to Equations (1) and (2) was determined as a function of filtration time *t* for each filtration pressure and temperature combination (*p*, *ϑ*) (Figure 6 and Figure 7). In dead-end mode, the thickness increases with time for all (*p*, *ϑ*) in the hollow fibre because more retained proteins accumulate on the membrane surface. At the beginning of filtration, for the first 35 min, the deposit layer height is similar for all three filtration pressures. A small pressure-dependence can only be observed at *ϑ* = 45 °C. The deposit layer height therefore seems to be almost pressure-independent for the first 35 min (Figure 6c).

After *t* = 35 min, the pressure influence on the deposit layer height is more pronounced for all measured temperatures: At *ϑ* = 15 °C and *ϑ* = 22 °C, thicker depositions were measured almost continuously with increasing pressure. However, the differences are small, and the filtration pressure at the two lower temperatures seems to have only a minor effect on accumulation.

The filtration at *p* = 0.75 bar and *ϑ* = 45 °C consistently yielded the highest deposit thickness for the whole filtration. The deposit height at the highest filtration pressure *p* = 1.25 bar at *ϑ* = 45 °C is very similar compared to the lowest filtration pressure *p* = 0.25 bar (Figure 6c). One possible explanation is that, at *p* = 1.25 bar and *ϑ* = 45 °C, the protein deposition is highly compressible and thus the same deposit thickness was measured as for *p* = 0.25 bar.

Looking at the protein concentration in Figure 6d measured on the membrane surface as a function of temperature, it can be clearly seen that the higher the temperature, the higher the casein concentration. At *ϑ* = 15 °C an average casein concentration of *c_protein_* = 88.3 g m^−2^ was measured for all three pressures. The protein concentration at *ϑ =* 15 °C was independent of pressure. With increasing temperature up to *ϑ* =45 °C the casein concentration raises at a pressure of *p* = 0.25 bar to *c_protein_* = 133.8 g m^−2^. At a pressure of *p* = 0.75 bar the concentration increase to *c_protein_* = 144.9 g m^−2^ and at a pressure of *p* = 1.25 bar to *c_protein_* = 153.3 g m^−2^. Overall, the pressure-dependence can only be clearly observed at *ϑ* = 45 °C as (Figure 6c,d).

To show the effect of temperature, *h*_d_ and *c*_Protein_ were plotted for each of the three pressure levels (Figure 7). At lower pressures, the deposit layer height is more temperature-dependent than at *p* = 1.25 bar.

Similar deposit layer thicknesses were observed for the investigated pressures, indicated by the constant scale in the figures. One possible explanation is that the deposit layer is highly compressed at *p* = 1.25 bar, which results in a more compact layer with similar height.

At pressures of *p* = 0.25 bar and *p* = 0.75 bar, the deposit layer formation shows a significant temperature dependence, especially for *t* > 35 min (Figure 7a,b). At the lowest pressure, the deposit height is most pronounced at *ϑ* = 45 °C and least pronounced at *ϑ* = 15 °C. At temperatures *ϑ* > 40 °C milk tends to undergo structural changes [49], which leads to stronger bonds between the milk proteins and the formation of agglomerates, which intrinsically influences compressibility. In the case of aggregation, the deposit layer would grow faster when compared to smaller molecules [20].

The measurements at *p* = 1.25 bar show almost a temperature independence for *t* < 75 min (Figure 7c). A small difference in deposit height can only be measured for *t* > 100 min. Concluding, the influence of temperature seems to be less at higher pressures. The pressure probably is so high that structural changes of the milk proteins dominate the process leading to almost no influence of temperature on the overall deposit layer height.

In contrast, the temperature dependence can be seen when the protein concentration is plotted as a function of pressure (Figure 7d). At *ϑ* = 15 °C the casein concentration constantly remains at *c_protein_* = 88.3 g m^−2^. Similarly, at *ϑ* = 22 °C the casein concentration increases linearly with increasing pressure. Overall, increasing protein concentrations with increasing temperature could be measured at all three pressures. The temperature dependence can be explained by the viscosity change of the skim milk [1]. As temperature rises, viscosity decreases, resulting in a higher flux and thus stronger particle deposition forces [37]. Consequently, more deposit should be formed. The casein to whey protein ratio is approximately 11:1 at *ϑ* = 15 °C and *ϑ* = 22 °C, whereas at *ϑ* = 45 °C, the ratio increases to 19:1 on average (Figure 7d). Consequently, caseins are more abundant in the deposit layer than whey proteins.

By comparing the permeate mass *m* in Figure 5a with *h*_d_ in Figure 6 and Figure 7, a relation for both is observed: during the initial phase, mass increases as flux decreases. Consequently, the deposit height increases simultaneously. When a certain flux is reached, the curves flatten. It is assumed that, at the beginning of filtration, the proteins attach only loosely to each other and a strong increase in the deposit height is observed. Afterwards, the flux decreases, and the deposit height only slightly increases because casein micelles pack more densely. The accumulated integral permeate mass was modelled by *m*(*t*) = *a*_integral_ ∗ *t*^b,integral^ [25] which results in pressure and temperature dependent proportionalities and exponents (Table 3). According to Reihanian et al. [50], an almost exact square root dependence (*b*_integral_ = 0.5) is expected for a cake filtration The deviation of *b*_integral_ of 0.5 suggests that not mechanisms of pure cake filtration, but other factors such as pore blocking play a role for filtration performance.

In a first approach, the locally measured deposit height at *z* = 29 cm was modelled with the same empirical relation, *h*_d_(*t*) = *a*_local_ ∗ *t^b^*^,local^ to allow a direct comparison between integral mass and local deposit measurements.

The fit parameter *a*_local_ increases with temperature (Table 3). The fit parameter *b*_local_ decreases with pressure, apart from the experiments at *ϑ* = 22 °C. In comparison to the clear correlation between deposit layer formation and temperature, *h*_d_ does not always increase with pressure as compression and compaction play a significant role. The fit parameter *b*_local_ describes the time-dependence of deposit height measured by MRI. There is only a small difference for the different pressures, which indicates a similar deposit growth during dead-end filtration. Within the experimental accuracy, *b*_local_ (*z* = 29 cm) ≈ 0.5 for all measured pressures. However, the nature and composition of the deposit are assumed to be homogeneous in this approach. In addition, a small trend towards larger *b*_integral_ is observed in *m*(*t*) as a function of pressure. In the present MR image quantification, the deposit is considered as a homogeneous structure. It is not radially differentiated, which is a first attempt. The deposit was shown by MRI to consist of reversible and irreversible parts, and the composition is pressure-dependent [25]. Additional measurements and quantification tools are necessary to better characterise and quantify the accumulated proteins and their individual geometry and composition with the aim to comprehensively describe and model the deposits as a function of time and spatial coordinates.

### 3.5. Effects on Signal Intensity of the Last Voxel Right before the Selective Membrane Layer

**Figure 8:**
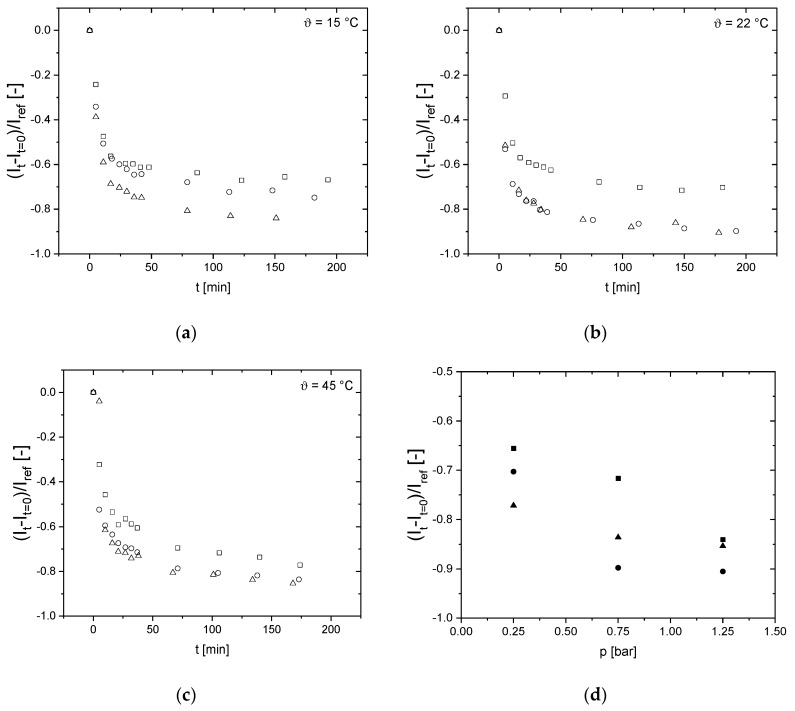
Reduced signal intensity of the voxel directly at the inner surface of the membrane as a function of filtration time *t* for (**a**) *ϑ* = 15 °C (**b**) *ϑ* = 22 °C and (**c**) *ϑ* = 45 °C by the concept described in [25] and measured for *p* = 0.25 bar (squares), *p* = 0.75 bar (circles) and *p* = 1.25 bar (triangles). (**d**) Comparing the reduced signal intensity at the selective layer of the membrane for *t* = *t*_max_ indicates that with increasing pressure and temperature the deposit found in MRI to be more compact. Temperature was set to *ϑ* = 15 °C (filled squares), *ϑ* = 22 °C (filled circles) and *ϑ* = 45 °C (filled triangles).

Looking into the details of the images, the following becomes evident: Directly at the inner surface of the membranes, a lower signal intensity at higher feed pressures shows the pressure dependence of the deposit and its structure. At the highest measured feed pressure of *p* = 1.25 bar, the signal intensity is the lowest not only at the membrane surface but also over the entire deposit layer. The MR signal intensity in each time step *I*_t_ is corrected by the reference image before the filtration (*t* = 0 min, *I*_t=0_) due to a susceptibility difference at the membrane wall and then normalized by the feed signal intensity in the middle of the membrane’s lumen *I*_ref_ resulting in (*I*_t_-*I*_t=0_)/*I*_ref_ (Figure 8): Directly at the membrane surface, the signal intensity is shifted towards lower values for higher filtration pressures. This indicates that the deposition becomes more compact. The deposit thickness is almost the same for all pressures during the initial phase of filtration and starts to differ with increasing *t* (Figure 8). Additionally, a temperature dependence is evident: the compactness increases with temperature indicating a better deformability of the proteins at higher temperatures.

These findings are in accordance with other observations: A higher convective flux and compression of the deposit may exist at larger pressures, which may change the nature of the deposit and its reversibility [25,51]. When the reduced signal intensity at the last measured voxel right at the membrane’s selective layer at *t* = *t*_max_, a pressure and temperature dependence can be observed. Yet, by measuring on the microscale with MRI, multiple effects need to be considered regarding the experimental uncertainty that can result in the shift of the reduced signal intensities. The signal intensity of the last voxel is affected by an uncertainty because multiple effects need to be considered. The possibility of a partial volume effect and other effects such as susceptibility differences are predominant and the other voxels toward the inside of the membrane lumen are left out of consideration. Additionally, a change or transition of the deposit layer to a gel layer in this pressure and temperature region might occur that contributes to a non-monotonic behavior of the reduced signal intensity. Yet, a dependence of the signal intensity, i.e., deposit layer, on pressure and temperature can be observed.

### 3.6. Impact of Milk Protein Concentration on the Height of the Deposit Layer

**Figure 9:**
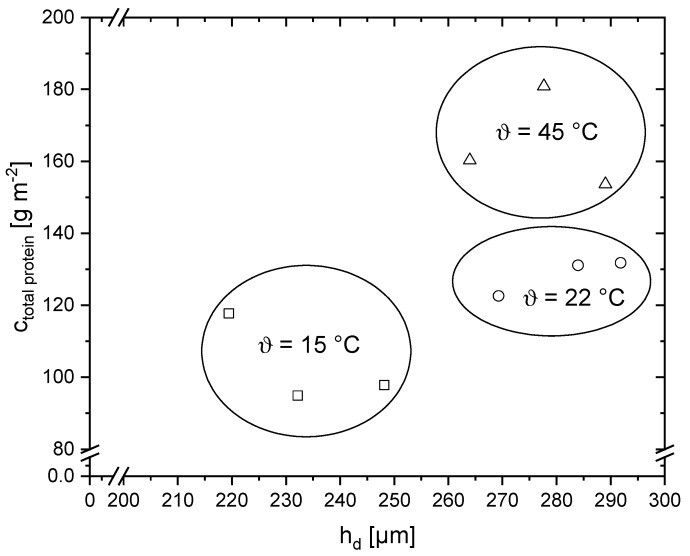
Total protein concentration *c*_total protein_ measured by RP-HPLC as a function of *h*_d_ measured by MRI for the three temperatures *ϑ* = 15 °C (squares), *ϑ* = 22 °C (circles) and *ϑ* = 45 °C (triangles).

To gain a better understanding how temperature and pressure affect the deposit’s chemical and structural composition as well as height, the total protein concentration measured by RP-HPLC was plotted as a function of *h*_d_ by MRI (Figure 9).

The plot of total protein concentration versus deposit height shows that higher deposit layers are generally associated with higher protein concentrations. When temperature was increased from *ϑ* = 15 °C to *ϑ* = 22 °C, both the deposit height and the milk protein concentration increase. In comparison to that, the deposit height at *ϑ* = 45 °C does not increase further, while the protein concentration increased from approximately 130 to around 170 g m^−2^. The deposit layer at *ϑ* = 45 °C is concluded to be more compact or dense than at *ϑ* = 15 °C and *ϑ* = 22 °C.

## 4. Conclusions

A method based on magnetic resonance imaging (MRI) to characterize deposit layer formation during milk protein fractionation was presented. Furthermore, a correlation between the time-dependent deposit and integral filtration parameters was found for dead-end skim milk filtration. Skim milk filtration was monitored as a function of pressure and temperature, which results in a deeper understanding of the (local) filtration and fouling mechanisms measured *in-situ*, supporting the pursuit of an optimised milk protein fractionation. The protein concentration and the height of the deposit correlate. Moreover, pressure affects the filtration behaviour on a microscopic length scale. MRI methods will be developed further to allow *in-situ* measurements in cross-flow filtration mode and to assess whether the reduced height of deposits can also be related to variations in processing conditions such as cross-flow velocity and transmembrane pressure as a function of axial position along the membrane.

## Figures and Tables

**Table 1:** Composition of the skim milk.

Content or Property of the Redispersed Skim Milk	Value Determined by Manufacturer (%w/w)	Measured Value
Casein protein	2.8	
Whey protein	0.7	
Fat	0.06	
Lactose	5.61	
Ash	0.8	
pH		6.6
*ϑ*		22 °C
η_Water_		0.992 mPa s
η_Milk_		1.477 mPa s
η_Permeate_		1.106 mPa s
φ_Milk_		0.9977 kg dm^−3^

**Table 2:** Main RARE parameters for *in-situ* MR measurements of dead-end filtration of skim milk.

RARE Parameter	Value
*T*_R_	4 s
$\tau_{E}$	5.5 ms
pixel size Δ*x* = Δ*y*	32.5 µm
RF	2
No. of averages	1
Slice thickness along *z*	3 mm
Time for the measurement	5 min 8 s
Encoding order	Centric
Partial Fourier Factor (Phase)	1.3

**Table 3:** Fit parameters *a* and *b* of modelling the permeate mass and deposit height by *m*(*t*), *h*_d_(*t*) = *a* ∗ *t*^b^.

*p* [bar]	*ϑ* [°C]	*a*_integral_ [-]	*b*_integral_ [-]	*a*_local_ [-]	*b*_local_ [-]
0.25	15	0.32	0.48	15.9	0.52
	22	0.30	0.52	18.8	0.51
	45	0.28	0.56	24.3	0.47
0.75	15	1.29	0.37	18.3	0.51
	22	0.60	0.44	21.2	0.50
	45	0.80	0.44	31.8	0.43
1.25	15	0.089	0.67	16.6	0.54
	22	0.46	0.52	19.8	0.52
	45	0.50	0.52	20.6	0.51
